# The Medical Session

**Published:** 1900-10-06

**Authors:** 


### The Hospital

A Journal of

The Medical Sciences and Hospital Administration.

Vol. XXIX.—No. 732.] Edited by Sir Henry Burdett, K.C.B., and Solomon C. Smith, M.D., M.R.C.P. [October 6, 1900.

### The Medical Session.

The pathetic exclamation of Abernethy, "What is to become of you all?" uttered as his eyes fell upon the crowded benches of his lecture room, will no doubt be recalled, to persons of pessimistic tendency, by the recollection that many similar sets of benches were equally crowded on Monday last, and will remain so, day after day, for the remainder of the medical session. The popularity of the profession as a pursuit to be embraced by young men does indeed fluctuate somewhat from year to year, but has never either so fallen or threatened to fall as to justify any anxiety lest the public should be deprived of medical or surgical assistance in time of need. The work of a doctor, indeed, is of a character which appeals strongly to the imaginations of the young. In how many domestic incidents does he appear upon the scene as a *deus ex machinâ*, dissipating alarm, relieving pain, rescuing from danger, and winning, at least until the trouble is forgotten, the gratitude and praise of all who either witness or benefit by his ministrations. Such a part as this can hardly fail to commend itself to a generous boy, or to excite a desire, in the minds of those whose home life has brought with it experience of illness or accident, to fill in time a position of similar usefulness and of similar popularity. In spite of pessimism, moreover, it may reasonably be believed that the prospects of a young man always depend more upon himself and upon his own qualities than upon the vocation which he selects; and that he who fails as a doctor would in all probability have failed with equal certainty as a barrister or an attorney. There are few professions, again, which afford scope for such varied activity as the medical. The philosophically-minded student may devote himself to research, and spend his days in the seclusion of the laboratory. The man of literary culture will be at home in the more refined circles of great cities; the man of rural or sporting tastes will seek for the opportunities afforded by country life and practice; the adventurous will find their proper sphere in the Navy or Army, or in the exploration of as yet only partially known countries; and, in short, the activities of the medical profession are of such a varied nature as to afford almost an infinite number of holes, of every variety of depth and outline, into which the human pegs calculated to fill them may be placed with advantage at once to themselves and to the community. The one mental quality which we should regard as antagonistic to success as a doctor is that known as the "business" or "commercial" instinct. A young man in whom this is powerful or predominant should be advised to seek more legitimate outlets for its profitable employment. If he engage in medical practice he will probably fail to cure his patients; and in that case, unless he should devote himself to some of the many forms of professional quackery, he will be likely, in a short time, no longer to have patients to cure.

It is a matter of grievous complaint among some people, and more especially among practitioners who have been but slenderly favoured by fortune, that the profession does not hold its proper place in the estimation of the public. The truth of this complaint must be admitted, if by "proper place" we mean that which the aims and the work of the highest class of physicians or surgeons should command. The admission, thus guarded, leaves it as an open question whether the complainants themselves do not, after all, obtain pretty nearly what they deserve. Public opinion, in the long run, is usually guided by an almost instinctive perception of justice and of desert; and if it be true, as we think it is, that individual practitioners often hold among their *clientèle* a highly desirable or even enviable position, the result of close personal knowledge and observation of their lives and conduct, while at the same time the profession as a whole is less favourably regarded, there must somewhere be an explanation of what at first sight appears to be anomalous. We are much disposed to seek for this explanation in an instinctive recognition of the utter inadequacy of the preliminary education which is required from medical students, and which, indeed, as a preparation for the study of a learned profession, is nothing more than a portentous sham. The schoolmasters and schoolfellows of the future doctors, the parson of the parish and the other people of culture around their homes, know the mental calibre and the sort of training which are regarded by all concerned as a sufficient equipment for entrance upon medical study. Hundreds of men are admitted as medical students whose natural powers are in no way above the average, and whose education has been little better than a farce; who have never been taught anything sufficiently well to teach them how to learn. If the heads of the profession do not insist upon adequate mental training as a preliminary to entrance upon special study, they will be taken, by public opinion, to admit that such training is not necessary for the work which practitioners will be called upon to do, and the respect which is paid to learning will no longer be regarded as due to them. If we add to this that the examining bodies seldom (if ever) reject a candidate as totally unfit for the profession, but allow an incompetent youth to come up again and again, until the examiners are weary of seeing him, and at last, like the unjust judge in Scripture, admit him by reason of his importunity, we have, we think, a sufficient reason why the position accorded to the most distinguished members of the profession, who are judged by their personal works, is not extended to the majority, who are only judged of collectively.

